# Prognostic Value of ^18^F–FDG–PET Parameters in Patients with Small Cell Lung Cancer: A Meta-Analysis and Review of Current Literature

**DOI:** 10.3390/diagnostics11020174

**Published:** 2021-01-26

**Authors:** Tine Nøhr Christensen, Per Kragh Andersen, Seppo W. Langer, Barbara Malene Bjerregaard Fischer

**Affiliations:** 1 Department of Clinical Physiology, Nuclear Medicine & PET, Rigshospitalet, 2100 Copenhagen Ø, Denmark; Malene.fischer@kcl.ac.uk; 2 Cluster for Molecular Imaging, University of Copenhagen, 2200 Copenhagen N, Denmark; 3 Section of Biostatistics, University of Copenhagen, 1014 Copenhagen K, Denmark; pka@biostat.ku.dk; 4 Department of Oncology, Rigshospitalet, 2100 Copenhagen Ø, Denmark; Seppo.Langer@regionh.dk; 5 Department of Clinical Medicine, University of Copenhagen, 2200 Copenhagen N, Denmark; 6 PET Centre, School of Biomedical Engineering and Imaging Science, King’s College London, London SE1 7EH, UK

**Keywords:** FDG–PET/CT, small cell lung cancer, prognosis, SUV_max_, metabolic tumor volume

## Abstract

Many studies have suggested a prognostic value of one or several positron emission tomography (PET) parameters in patients with small cell lung cancer (SCLC). However, studies are often small, and there is a considerable interstudy disagreement about which PET parameters have a prognostic value. The objective of this study was to perform a review and meta-analysis to identify the most promising PET parameter for prognostication. PubMed^®^, Cochrane, and Embase^®^ were searched for papers addressing the prognostic value of any PET parameter at any treatment phase with any endpoint in patients with SCLC. Pooled hazard ratios (HRs) were calculated by a random effects model for the prognostic value of the baseline maximum standardized uptake value (SUV_max_) and metabolic tumor volume (MTV). The qualitative analysis included 38 studies, of these, 19 studies were included in the meta-analyses. The pooled results showed that high baseline MTV was prognostic for overall survival (OS) (HR: 2.83 (95% confidence interval [CI]: 2.00–4.01) and progression-free survival (PFS) (HR: 3.11 (95% CI: 1.99–4.90)). The prognostic value of SUV_max_ was less pronounced (OS: HR: 1.50 (95% CI: 1.17–1.91); PFS: HR: 1.24 (95% CI: 0.94–1.63)). Baseline MTV is a strong prognosticator for OS and PFS in patients with SCLC. MTV has a prognostic value superior to those of other PET parameters, but whether MTV is superior to other prognosticators of tumor burden needs further investigation.

## 1. Introduction

Small cell lung cancer (SCLC) is an aggressive cancer, and most patients present at an advanced stage [1]. Treatment options are limited. Patients with limited disease (LD) are treated with concomitant thoracic radiotherapy and platin-based chemoradiotherapy. Patients presenting at an advanced stage (extensive disease; ED) are treated with palliative platin-based chemotherapy. Up to 40% of patients do not achieve objective response to first-line therapy [2], but even when objective response is achieved, it is often followed by a quick and fatal relapse, and overall survival (OS) is poor [2]. The introduction of immunotherapy for first-line treatment and for treatment of relapse gives hope for an improved clinical outcome [3,4,5].

2-Deoxy-2-[^18^F]fluoro-D-glucose (FDG) positron emission tomography (PET)/computed tomography (CT) has an established role in the staging of SCLC with a sensitivity approximating 100% and a specificity exceeding 90% [6,7]. Compared with CT, FDG–PET/CT causes stage migration in up to 40% of patients, thus having a great impact on treatment choice [8]. FDG–PET/CT for early or final response evaluation seems feasible [9]; however, the role of FDG–PET/CT after therapy has not been proven to be superior to that of CT [10]. Several studies have shown a prognostic value of FDG–PET/CT, but studies are inconsistent in regard to which parameters have a prognostic value and cutoff values differ [9]. Better prognostication in order to personalize the aggressiveness of the treatment course and surveillance after the end of treatment is warranted.

In this study, we present an overview of all published studies of the prognostic value of FDG–PET parameters before, during, and after treatment in patients with SCLC, including quantification by a meta-analysis of baseline PET parameters, in order to identify the most promising PET parameter(s) for prognostication.

## 2. Materials and Methods

### 2.1. Eligibility Criteria

Studies concerning the prognostic evaluation of any FDG–PET parameter in patients with SCLC were eligible. Studies were not selected based on the stage of SCLC, treatment, or other clinical characteristics.

FDG–PET performed at any phase of the disease was accepted: before treatment, during treatment, after the end of treatment, and during follow-up.

Any PET parameter was accepted (uptake values, metabolic tumor volumes, and their combinations).

PET parameters within any anatomical region were accepted (within primary tumor, lymph nodes, metastases, and their combinations).

Any prognostic endpoint was accepted (progression-free survival (PFS), distant failure, time to progression, OS, and so forth).

#### 2.1.1. Search Strategy

A search was performed in PubMed^®^, Cochrane Library, and Embase^®^ on 24 September 2020. MeSH^®^ terms were used in PubMed^®^ and Cochrane Library, and Emtree^®^ terms in Embase^®^, in combination with the search of keywords.

The search in PubMed^®^ and Cochrane Library was constructed as follows: ((carcinoma, small cell lung [MeSH terms]), OR (SCLC)) AND ((positron emission tomography [MeSH Terms]) OR (positron emission tomography) OR (PET)) AND ((18f fluorodeoxyglucose [MeSH Terms]) OR (fluorodeoxyglucose) OR (FDG)) AND ((prognosis) OR (prognosis [MeSH Terms])).

The search in Embase^®^ was constructed as follows: ((small cell lung cancer/) OR (SCLC.mp)) AND ((positron emission tomography/) OR (PET.mp) OR (positron emission tomography.mp)) AND ((fluorodeoxyglucose f 18/) OR (fluorodeoxyglucose/) OR (fluorodeoxyglucose.mp) OR (FDG.mp)) AND ((prognosis/) OR (prognosis.mp)).

#### 2.1.2. Study Selection

The papers identified by the database search were screened for inclusion. Reviews, cases, meta-analyses, letters, preclinical studies, trial notes, and studies in languages other than English were excluded. Reference lists from the included studies were screened for additional records.

Studies with overlapping cohorts were included if different PET parameters or endpoints were addressed; otherwise, the study with the largest cohort was included.

Studies of baseline FDG–PET providing hazard ratio (HR) and 95% confidence intervals (CI) for PFS or OS or sufficient data to extract HR and 95% CI were included in the meta-analysis.

### 2.2. Data

Clinical data, PET parameters, and prognostic data were extracted from the identified records.

The prognostic value of PET parameters at variant time periods in regard to treatment was qualitatively described. The independent prognostic value of PET parameters was compared with that of clinical parameters in studies providing multivariate analysis.

Risk of bias in the studies was assessed by six domains using the Quality in Prognostic Studies (QUIPS) tool [11]. In the “study confounding” domain, inclusion of the covariates stage, age, and sex was assessed.

### 2.3. Statistics

The meta-analysis was performed for the baseline maximum standardized uptake value (SUV_max_) and baseline metabolic tumor volume (MTV) measured within the primary tumor (tSUV_max_, tMTV) or in the whole body (wbSUV_max_, wbMTV). Separate analyses were performed for the most common endpoints: OS and PFS.

HR and 95% CI from univariate analysis were collected. In studies not providing HR and 95% CI, data were extracted from Kaplan–Meier curves either with readable data points or combined with the available *p*-value and recalculated into the Cox model. In studies providing HR for continuous values of SUV_max_ or MTV, data points for individual patients were extracted from Kaplan–Meier curves when available, or a Cox model was reconstructed for the dichotomized SUV_max_ or MTV. If individual data points were not available, the difference of the median value in the high group and the low group was applied, and HR was estimated for the dichotomized PET parameters. See Supplementary Materials File S2 for further details on the reconstruction of data.

Meta-analyses were performed using the functions “metagen,” “forest,” and “funnel” in the R package “meta” version 4.9-1 (R Foundation for Statistical Computing, Vianna, Austria). Due to the inherent heterogeneity of the studies owing to differences of study designs and definitions of PET parameters, random effects models were used. Forest plots and pooled HR and 95% CI were generated. HR greater than one implies worse survival for patients with larger PET parameters. Heterogeneity between the studies was evaluated by I^2^ and tau^2^ statistics. Funnel plots were constructed to identify the presence of publication bias.

## 3. Results

**Figure 1:**
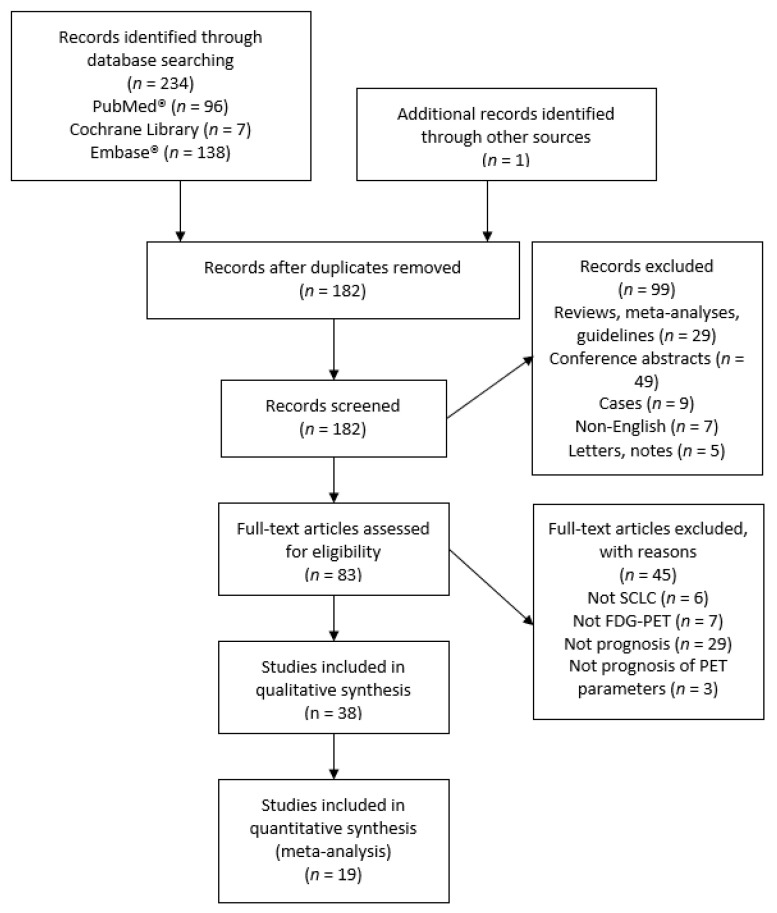
Prisma flowchart of included and excluded studies. SCLC: small cell lung cancer; FDG–PET: 2-deoxy-2-[^18^F]fluoro-D-glucose positron emission tomography.

The search on PubMed^®^, Cochrane, and Embase^®^ resulted in 181 individual records. After excluding 144 records, 37 studies were included in the qualitative review. One additional study was identified through screening the references of the included studies. Nineteen studies were included in the quantitative meta-analysis. The identification process and reasons for exclusion are illustrated in Figure 1. Four studies had a partial overlap of patient cohorts with one other study each [12,13,14,15]. They were all included in the qualitative review as their designs differed. The smallest study of Oh et al. [13] was excluded from the meta-analysis in favor of a larger study [12]. The study of Kim et al. [14] was excluded from the meta-analysis due to insufficient data.

From the 38 included studies, 30 studies addressed the prognostic value of baseline PET parameters. Post-treatment PET parameters were evaluated in 7 studies, the prognostic value of changes in PET parameters was evaluated in four studies, and further three studies evaluated the prognostic value of PET parameters in different timings, because the patient cohorts consisted of patients who had performed PET before or after treatment or before and during therapy.

The 38 studies present 73 different approaches of measuring PET parameters. Table 1 defines the 73 different PET parameters.

### 3.1. Quality of the Studies

**Figure 2:**
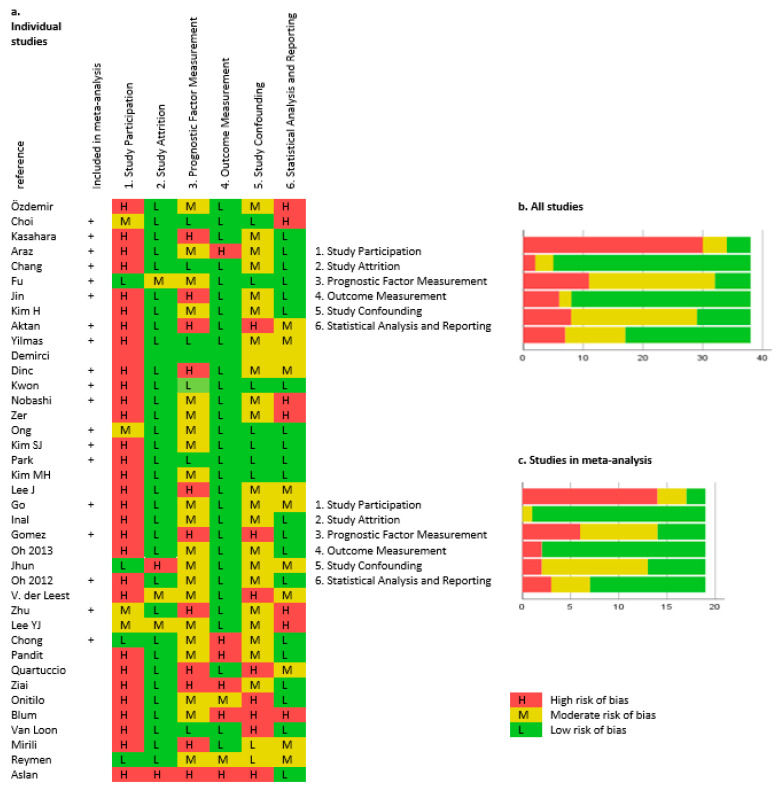
Risk of bias according to the Quality in Prognostic Studies (QUIPS) tools. Individual studies are shown in (**a**), results from all studies in (**b**), and results from studies included in the meta-analysis in (**c**).

Figure 2 presents the risk of bias in the included studies evaluated using the QUIPS tool. There was a high risk of bias in “study participation,” reflecting a retrospective design of 35 of the included studies. Available PET and medical records were inclusion criteria in most studies, causing inclusion of as little as 13% of all SCLC patients from the recruiting period [16].

“Prognostic factor measurement” had moderate or high risk of bias in 32 studies, including 14 studies in the meta-analysis. The risk of bias for the prognostic factor measurement was often caused by the use of optimal cutoff (*n* = 6), median cutoff (*n* = 17), or no available information of which cutoff was used (*n* = 4). PET acquisition and definition of PET parameters rarely contributed to bias. Few studies did not provide sufficient data, and in one study, baseline PET performed up to 4 months prior to the start of treatment was assessed [17].

### 3.2. Qualitative Analysis: Prognostic Value of Baseline PET Parameters

Results from the 30 baseline studies are presented in Table 2. Each study included 8 to 344 patients.

#### 3.2.1. Baseline SUV

Baseline SUV_max_ was addressed in 28 studies, but only seven studies showed a significant prognostic value of SUV_max_ for OS and/or PFS [18,19,20,21,22,23,24].

**Figure 3:**
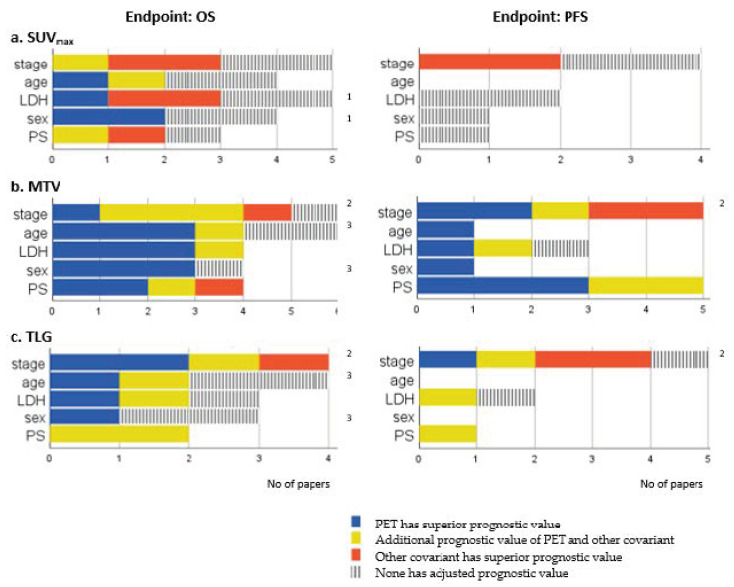
Comparisons of PET parameters and other covariates included in the multivariate analysis. Number of papers showing either superior (blue), additional (yellow), inferior (red), or no prognostic value (grey) of adjusted SUV_max_ (**a**), MTV (**b**), and TLG (**c**) compared with the five most frequently used covariates. ^1^ Özdemir accounted twice due to different results in subgroups, ^2^ Nobashi accounted twice due to different results in subgroups, ^3^ Choi accounted twice due to different results in subgroups.

Twelve studies included baseline SUV_max_ in a multivariate analysis. In five studies, SUV_max_ were independently prognostic for OS [18,19,20,21,25]. No study showed an independent prognostic value for PFS [12,25,32,33]. Compared with other covariates included in the multivariate analysis, an additional independent or superior prognostic value of SUV_max_ to stage, age, blood lactate dehydrogenase (LDH), sex, and performance status (PS) was sporadic (Figure 3a).

Other uptake parameters than SUV_max_ have been evaluated for prognostic value. SUV_peak_ [26,29] and SUV_mean_ [16,26,34,35] did not show a significant prognostic value in any studies. MeanSUV_max_ (mean of SUV_max_ from all lesions) was prognostic for OS and PFS in one of four studies [40]. Lesser-used PET parameters were addressed in one study each, all showing a prognostic value: MeanSUV_mean_ (mean of SUV_mean_ from all lesions) [23], SUV_max_ corrected for blood glucose level (SUV_max_(glu)) [27], and SUV_max_ corrected for liver-FDG uptake (SUV_max_(liver)). However, in contrast to other uptake parameters, high SUV_max_(liver) was associated with a better prognosis (HR by univariate analysis: 0.31) [36].

Three uptake parameters showed an independent prognostic value for OS and/or PFS in one study each: wb-meanSUV_max_ (HR for OS: 3.74; HR for PFS: 2.25) [40], t-SUV_max_(glu) (HR for PFS: 3.38) [27], and tSUV_max_(liver) (HR for OS 0.194) [36].

#### 3.2.2. Baseline MTV

Baseline MTV was addressed in univariate analysis in 13 studies. All studies showed significant prognostic results for OS, PFS, and/or distant failure.

Absolute threshold was the most frequently used delineation method. Large MTV2.5 was prognostic for lower OS in four of five studies [16,21,23,27] and for lower PFS in four of five studies [16,21,23,29]: MTV2.5 measured throughout the whole body (wbMTV2.5) was prognostic for OS and PFS in two of two studies [21,23]. MTV2.5 measured within the primary tumor (tMTV2.5) was prognostic for OS in one of two studies [27], but not for PFS [16,27]. MTV2.5 measured within the primary tumor and lymph node metastases (tnMTV2.5) was prognostic for PFS in two of two studies [16,29], and for OS in one of two studies [16].

MTV3.0 throughout the whole body (wbMTV3.0) had a prognostic value for OS in four of four studies [12,13,18,28], though partial cohort overlap of two of the studies should be noticed [12,13]. wbMTV3.0 had a prognostic value for PFS in two of three studies [13,28]. MTV3.0 measured in the primary tumor (tMTV3.0), measured in all intrathoracic tumors, or in the hottest tumor did not show a significant prognostic value [13,18].

MTV with relative thresholds of 40% or 42% of SUV_max_ (MTV40; MTV42) showed a prognostic value for OS and PFS in one [32] of two studies. Ong et al. [34] showed a prognostic value of tMTV42 for distant failure, but not for OS or PFS.

Software-delineated MTV (MTV_software_) was prognostic for OS in two of two studies [26,35]. Both studies used a patient-specific SUV threshold for delineation based on SUV in the liver. The prognostic value of MTV_software_ for PFS has not been investigated.

Results from multivariate analysis of baseline MTV were available from 14 studies, accounting for the above 13 studies and the study of Zer et al. [33] that had only published results from multivariate analysis.

Baseline MTV had an independent prognostic value for OS (HR: 1.001–16.7) and/or PFS (HR: 1.8–6.11) in 12 of 14 studies.

PET parameters and clinical parameters were comparable for OS in 10 studies, and for PFS in 8 studies. Figure 3b gives an overview of the independent prognostic value of PET parameters and the most investigated covariates. MTV had an additional or superior prognostic value to stage [12,23,32,33,35], age [18,26,28,35], LDH [12,18,23,26], sex [18,26,28], and PS [12,13,16,28] in most studies. Only three studies identified a clinical covariate with a superior prognostic value to MTV: stage [32], stage and treatment response [29], and PS, chemotherapy and number of extrathoracic metastases [13].

#### 3.2.3. Baseline PET Parameters Combining SUV with Tumor Volume

Eleven studies addressed total lesion glycolysis (TLG; the product of MTV and SUV_mean_ within MTV). In nine studies, TLG provided similar results as MTV [16,18,21,23,27,29,32,33,35]. However, in the studies of Araz et al. [26] and Ong et al. [34], TLG did not show a prognostic value, whereas MTV did.

TLG had an independent prognostic value for OS (HR: 1.0003–11.19) in six studies [16,18,23,32,33,35] and for PFS (HR: 3.2–12.48) in three [16,23,32]. Stage was the most frequently investigated clinical parameter in addition to TLG. TLG had an additional or superior prognostic value to stage for OS [23,32,33,35], but the results applied only for one subgroup in the study of Nobashi et al. [1]. Other clinical parameters were sporadic included in the multivariate analysis (Figure 3c).

The sum of SUV_max_ from all lesions (sumSUV_max_) was addressed in two studies, both showing a prognostic value for PFS and OS [14,37]. Baseline sumSUV_max_ had an independent prognostic value (OS: HR: 2.676–3.970; PFS: HR: 2.219–2.296) in both studies. SumSUV_max_ was a stronger prognosticator for OS than stage and sex.

### 3.3. Qualitative Analysis: Prognostic Value of Post-Treatment PET Parameters

Table 3 presents results from seven studies addressing the prognostic value of FDG–PET/CT after treatment. The studies included 22–164 patients each. The majority of studies investigated the prognostic value of PET within 4 months after the end of treatment, although Pandit et al. [41] included patients up to 4 years after treatment.

In five studies, either SUV_max_ [29,41,43], SUV_peak_ [29], wbSUL_peak_ [43], presence of PET-positive lesions [41,43,44,45], MTV2.5, or TLG2.5 [29] showed a prognostic value. Two studies, including the largest study, did not find a significant prognostic value of any post-treatment PET parameter [36,42].

Multivariate analysis showed an independent prognostic value of post-treatment PET parameter in two of three studies: tnMTV2.5 was independently prognostic for PFS (HR: 2.8 (95% CI: 1.5–5.2), *p* = 0.001) in addition to initial stage and response by The Response Evaluation Criteria in Solid Tumors (RECIST) [29]; sum-wbSUL_peak_ and presence of PET-positive lesions were independently prognostic for OS and/or PFS (HR 1.046) [43].

### 3.4. Qualitative Analysis: Prognostic Value of PET Parameter Change, Early and Final Response Evaluation

Results from four studies evaluating the prognostic value of a PET parameter change from baseline PET to PET during or after the end of treatment are presented in Table 4.

Van Loon et al. showed a prognostic value of early response measured as the reduction of MTV after one cycle of chemotherapy, despite a small study size (*n* = 15) [46]. The PET parameter change from baseline to the end of therapy (i.e., final response evaluation) had a prognostic value in three of three studies; however, different PET parameters were tested: reductions of tSUV_max_, tSUV_peak_, tn-meanSUV_max_(liver), tSUL_peak_, and tnMTV2.5 were prognostic for PFS and/or OS [29,36,43]. Change of tSUV_max_(liver) and tnTLG2.5 did not show a prognostic value [29,36]. Reduction of SUV_peak_ had an independent prognostic value for OS over stage; however, for PFS, stage had an independent prognostic value over SUV_peak_ [29]. Reduction of tn-meanSUV_max_(liver) had an independent prognostic value for PFS in addition to LDH [36].

### 3.5. Qualitative Analysis: Prognostic Value of PET Parameters at Mixed Treatment Phases

Three studies investigated the prognostic value of PET parameters at mixed treatment phases (Table 5).

Two studies investigated a cohort mixed of patients who had baseline PET or post-treatment PET [47,49]. Both studies investigated SUV_max_, SUV_mean_, MTV, and TLG. Most analyses did not find any prognostic value. Mirili et al. [47] showed a prognostic value of SUV_max_ and MTV. Arslan et al. [49] found a prognostic value for OS of only TLG.

Gross tumor volume (GTV) used for radiotherapy planning based on pre- and post-chemotherapy PET/CT was prognostic for OS [48].

### 3.6. Quantitative Analysis: Prognostic Value of Baseline PET Parameters

#### 3.6.1. Baseline SUV_max_

Fourteen studies with a total of 1194 patients were included in the meta-analysis of the prognostic value of SUV_max_ with OS as endpoint. Nine studies with a total of 716 patients were included with PFS as endpoint. SUV_max_-cutoff for dichotomizing patients into two groups of high and low SUV_max_ ranged from 5.1 to 16. The cutoffs in the studies were median SUV_max_ (*n* = 7), optimal cutoff (*n* = 6), and recalculated median SUV_max_ from HR of a continuously increasing SUV_max_ (*n* = 3). Information of cutoff and definitions of SUV_max_ in the studies are available in Supplementary Materials File S1, Table S1.

**Figure 4:**
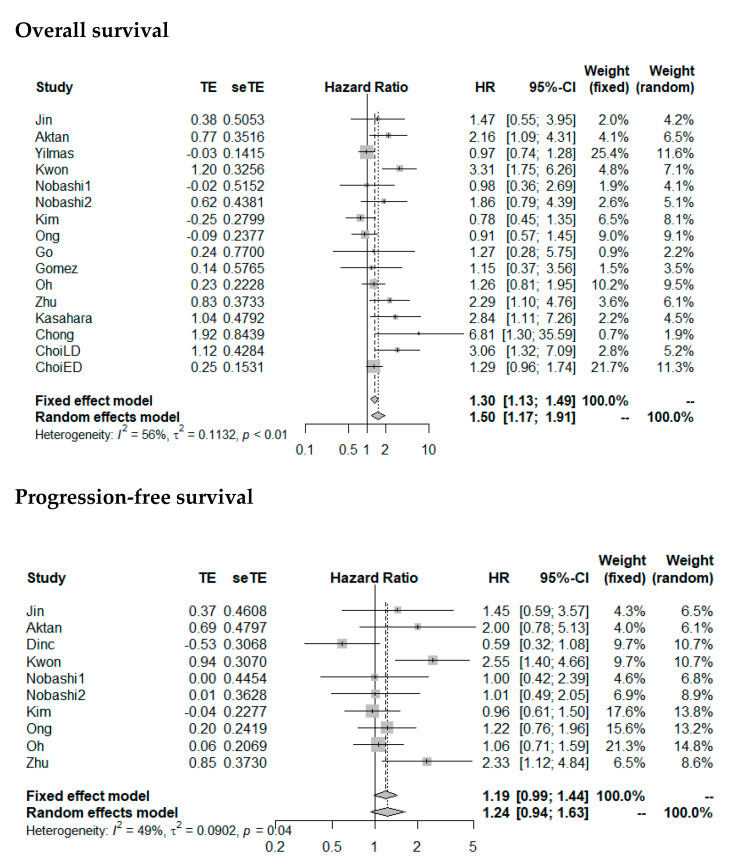
Forest plots of HRs of SUV_max_ for overall survival and progression-free survival. Nobashi1 refers to the results of the central type of SCLC. Nobashi2 refers to the results of the peripheral type of SCLC. ChoiLD refers to the result of LD-SCLC. ChoiED refers to the results of SCLC-ED.

**Figure 5:**
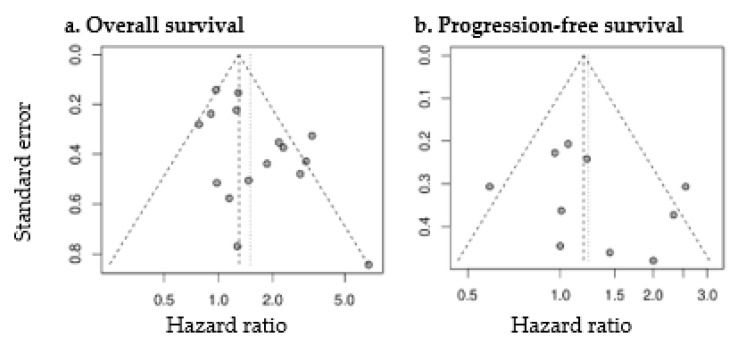
Funnel plots of studies assessing the prognostic value of SUV_max_ for OS (**a**) and PFS (**b**).

Random effects meta-analysis revealed a slightly increased HR for OS with large SUV_max_ (pooled HR: 1.50 (1.17–1.91), *p* = 0.001). SUV_max_ was not significantly prognostic for PFS (pooled HR: 1.24 (0.94–1.63), *p* = 0.13). Forest plots are presented in Figure 4. The heterogeneity between the studies was moderate (OS as endpoint: I^2^ = 56%, tau^2^ = 0.1132; PFS as endpoint: I^2^ = 49%, tau^2^ = 0.0902). Funnel plots showed a tendency toward asymmetry (Figure 5), which can be caused by interstudy heterogeneity or publication/reporting bias. The corresponding test for asymmetry was significant with OS as endpoint (*p* = 0.02), and not significant with PFS as endpoint (*p* = 0.35).

#### 3.6.2. Baseline MTV

Eleven studies with a total of 1015 patients were included in the meta-analysis of the prognostic value of MTV with OS as endpoint. Seven studies with a total of 627 patients were included in the meta-analysis with PFS as endpoint. MTV cutoff for dichotomizing patients in two groups with high and low MTV ranged from 21.45 (tMTV42) to 266.5 (wbMTV3.0). The cutoff in the studies was median MTV (*n* = 6), 75th percentile MTV (*n* = 1), or optimal cutoff (*n* = 3), as well as recalculated median MTV from HR using MTV as a continuous variable (*n* = 2). MTV was delineated with an absolute threshold in seven studies, with a relative threshold in three studies, and with a software-based method in two studies. Cutoffs and definitions of MTV in the studies included in the meta-analyses are available in Supplementary Materials File S1, Table S1.

**Figure 6:**
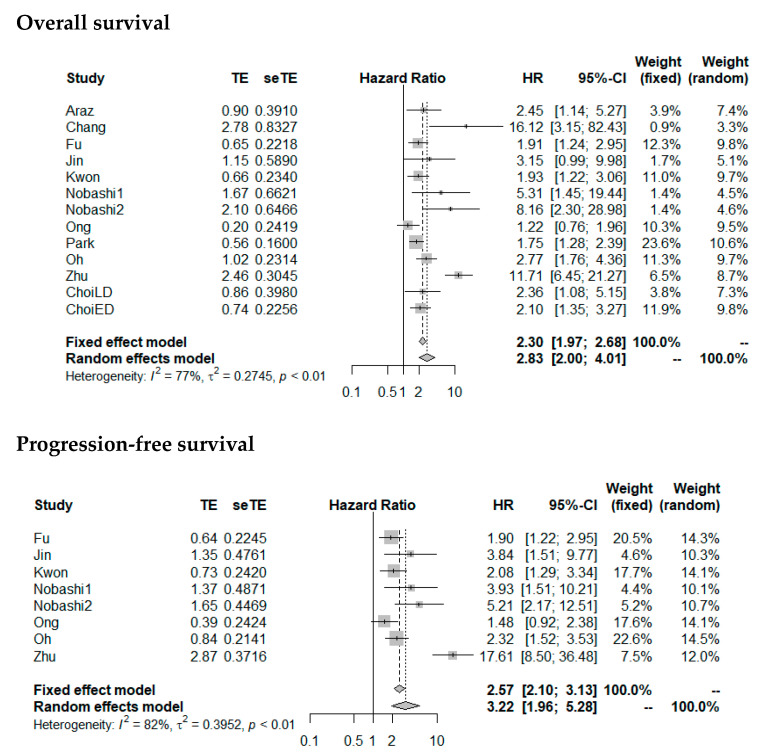
Forest plots of HRs of MTV for overall survival and progression-free survival. Nobashi1 refers to the results of the central type of SCLC. Nobashi2 refers to the results of the peripheral type of SCLC.

**Figure 7:**
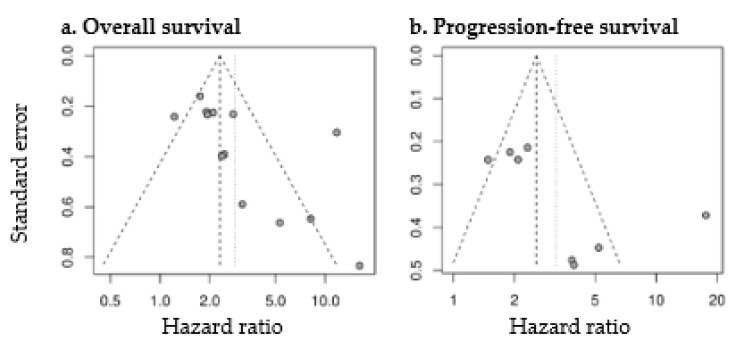
Funnel plots of studies assessing the prognostic value of MTV for OS (**a**) and PFS (**b**).

HR for OS and PFS was significantly higher with high MTV (pooled HR for OS: 2.83 (2.00–4.01), *p* < 0.0001; pooled HR for PFS: 3.22 (1.96–5.28), *p* < 0.0001). Forest plots are presented in Figure 6. The heterogeneity between the studies was high (OS as endpoint: I^2^ = 77%, tau^2^ = 0.2745; PFS as endpoint: I^2^ = 82%, tau^2^ = 0.3952). Funnel plots were asymmetric with larger HR for studies with lower precision (*p* = 0.04 for OS; *p* = 0.08 for PFS) (Figure 7), corresponding to the large interstudy heterogeneity, although publication bias is possible.

## 4. Discussion

This paper provides an overview and meta-analyses of PET parameters for prognostication in SCLC in order to identify the most valuable PET parameter for prognostication. From the available results, baseline MTV, regardless of the delineation method, performed well in individual studies, in the meta-analysis, and in multivariate analysis in the individual studies. MTV measured throughout the whole body performed better than MTV in the primary tumor. MTV was a stronger prognosticator than most clinical parameters and had an equal or additional prognostic value to stage. Baseline SUV_max_ did not show a convincing prognostic value in the qualitative analysis and showed only a slight prognostic value in the meta-analysis. TLG, combining MTV and SUV_max_, did not add a prognostic value to MTV. The compound parameter sumSUV_max_ showed promise in univariate and multivariate analyses, with either an additional or stronger prognostic value, compared with stage and objective response but was addressed in only two studies [14,37].

The prognostic value of PET parameters after treatment were addressed in seven studies and during treatment only in one study. Results were encouraging; however, due to the large variety of investigated PET parameters, it cannot be justified to appoint a superior PET parameter.

A previous meta-analysis on patients with SCLC established a small prognostic value of SUV_max_ for PFS (HR: 1.09) and OS (HR: 1.13) [50], similar to our results. However, a limitation to the meta-analyses of Zhu et al. is pooling of HR of high vs. low SUV_max_ with HR for continuously increasing SUV_max_ and inclusion of results from univariate and multivariate analyses. HR and 95% CI for a continuous increase is smaller than HR for a dichotomized parameter, affecting the weight of the studies in the pooled analysis. The meta-analysis of Zhu et al. included 1062 patients from 12 studies; however, more than 80% of the weight in the meta-analyses was based on data from one study with 59 patients [21]. Zhu et al. did not perform meta-analysis on MTV. In other cancers, including non-small cell lung cancer NSCLC [51], lymphoma [52], and head and neck squamous cell carcinoma [53], meta-analysis also demonstrated a superiority of MTV over SUV_max_. However, SUV_max_, but not MTV, was prognostic for event-free survival in a meta-analysis in patients with breast cancer [54]. It has previously been suggested that in advanced cancers, SUV_max_ may not be representative of tumor metabolism or tumor burden [55]. This may contribute to the different results seen in different cancers and could explain why wbMTV is a better prognosticator than SUV_max_ in SCLC. SUV_max_ represents the metabolism in one single voxel, whereas wbMTV reflects the entire tumor burden. In an aggressive cancer such as SCLC with a high metabolic activity in the vast majority of cases, it is likely that a prognosticator to even a higher extent needs to reflect the entire tumor burden to add value compared with that in other cancers.

Numerous PET parameters have been evaluated for prognostic value in patients with SCLC; however, to our knowledge, radiomic features have not yet been addressed in SCLC. Results from the prognostic value of radiomic features in patients with NSCLC have been inconsistent [56]. A validation study did not find an independent prognostic value of PET radiomics in NSCLC [57].

A comparison of the prognostic value of MTV with those of other parameters of tumor burden (i.e., volume measured by other imaging modalities or by the tumor, node, metastasis (TNM) staging system) would be relevant. Except for stage (ED vs. LD), LDH, and metastases, other parameters of tumor burden were not included in the papers. In NSCLC, a large validation study showed an independent prognostic value of MTV and TNM stage, and a combined index of MTV, TNM stage, and age improves the accuracy of OS prognosis [58].

This study has limitations. Meta-analyses often overestimate HR [59], and the possibility of publication bias must be considered. Funnel plots showed tendencies toward asymmetry, particularly for MTV, suggesting the presence of publication bias. However, interpretation of asymmetry tests should be done with caution when the included studies show large interstudy heterogeneity [60] and when the analysis includes censored data [61]. In these instances, which are both relevant for this meta-analysis, the asymmetry can be caused by heterogeneity. Most studies identified at least one PET parameter with a prognostic value, but in addition to their positive results, negative results from other PET parameters were also presented, and therefore, a small study effect does not seem obvious. However, the selection of which PET parameters are presented in each study may be biased. With 73 different approaches used to quantify PET parameters presented in the included 38 studies, and the fact that almost all studies identified at least one significant prognosticator, this calls for a concern for selective analysis reporting, favoring the presentation of PET parameters with positive results and, to a lesser extent, including PET parameters with negative results in the papers.

The risk of bias in the included studies was evaluated using the QUIPS tools. There was a high risk of bias within the domain “study participation” due to the retrospective design of 35 of 38 studies. Patients were included only if a baseline FDG–PET/CT was available, but the reasons for not having an available FDG–PET/CT were not given. The risk of bias in the domain “study confounding” was moderate to high in 29 of 38 studies and in 13 of 19 studies included in the meta-analysis. The prognostic value of adjusted PET parameters is more clinically relevant than an unadjusted prognostic value, and it has been recommended that the adjusted HR is used in meta-analyses [59]. However, different multivariate study designs were used in each study; thus a comparison of adjusted HRs in the meta-analysis would be highly biased. Additionally, the measurement of the PET parameters was associated with risks of bias, often caused by using a study-specific (optimal) cutoff for dichotomizing the patients into groups with high and low PET parameters.

The studies included in our meta-analysis showed a large interstudy heterogeneity. Apart from the different cutoff values for dichotomizing high vs. low SUV_max_ and MTV, differences in the included study populations, PET protocols, and definitions for PET parameters contributed to the heterogeneity. To accommodate the interstudy heterogeneity, random effects model meta-analyses were applied. We found a significant prognostic value of MTV for OS and PFS, and a lesser pronounced prognostic value of SUV_max_. A strong prognosticator should be able to prove its worth under a slightly varying condition, and the prognostic value of MTV may exist regardless of the delineation method, anatomical boundaries, and cutoff value, but it rather represents an increasing risk when MTV increases.

## 5. Conclusions

From these review and meta-analyses, we have identified baseline MTV as a strong prognosticator for PFS and OS in patients with SCLC. MTV has a prognostic value that is superior to those of other PET parameters, but whether MTV is superior to other prognosticators of tumor burden, such as stage and CT volumetrics, needs further investigation.

## Figures and Tables

**Table 1:** PET parameters. Definitions of PET parameters used in the included studies.

PET Parameters in Included Studies		Definition
**SUV: Standardized uptake value**		FDG uptake measured as the ratio of radioactivity in a region of interest (ROI) (voxel, cm^3^, tumor) and the mean radioactivity across the whole body
SUV_max_		The highest single-voxel SUV in a predefined ROI
	tSUV_max_	SUV_max_ in the primary tumor
	nSUV_max_	SUV_max_ in regional lymph node metastases
	mSUV_max_	SUV_max_ in distant metastases
	tnSUV_max_	SUV_max_ in the primary tumor and regional lymph node metastases
	wbSUV_max_	SUV_max_ in all malignant lesions throughout the whole body
	thoracicSUV_max_	SUV_max_ in intrathoracic malignant lesions (lung, pleura, mediastinum)
	extrathoracicSUV_max_	SUV_max_ in extrathoracic malignant lesions
	tn-meanSUV_max_	Average of SUV_max_ from primary tumor and regional lymph node metastases
	wb-meanSUV_max_	Average of SUV_max_ from each malignant lesion throughout the whole body
	wb-sumSUV_max_	Sum of all SUV_max_ from each malignant lesion throughout the whole body
	ΔtSUV_max_	Change of tSUV_max_ (e.g., from baseline to end of therapy)
SUV_peak_		Average of SUV within a small region of interest (e.g., 1 cm^3^) centered at the most active area in the tumor
	tSUV_peak_	SUV_peak_ in the primary tumor
	wbSUV_peak_	SUV_peak_ in all malignant lesions throughout the whole body
	ΔtSUV_peak_	Change of tSUV_peak_ (e.g., from baseline to end of therapy)
SUV_mean_		Average of SUV in an MTV; suffix indicates delineation method for MTV
	tSUV_mean2.5_	SUV_mean_ in MTV2.5 in the primary tumor
	tSUV_mean40_	SUV_mean_ in MTV40 in the primary tumor
	tSUV_mean42_	SUV_mean_ in MTV42 in the primary tumor
	nSUV_mean2.5_	SUV_mean_ in MTV2.5 in regional lymph node metastases
	nSUV_mean40_	SUV_mean_ in MTV40 in regional lymph node metastases
	mSUV_mean40_	SUV_mean_ in MTV40 in distant metastases
	wbSUV_mean2.5_	SUV_mean_ from all MTV2.5s throughout the whole body
	wbSUV_mean(software)_	SUV_mean_ from all MTV_software_ throughout the whole body
	thoracicSUV_mean(software)_	SUV_mean_ from MTV_software_ in intrathoracic malignant lesions (lung, pleura, mediastinum)
	wb-meanSUV_mean2.5_	Average of SUV_mean_ from each MTV2.5 throughout the whole body
SUL_peak_		SUV_peak_ in a 1 cm^3^ sphere normalized to lean body mass; recommended by PERCIST
	Wb-sumSULpeak	Sum of maximum 5 SUL_peak_’s throughout the whole body
	ΔtSUL_peak_	Change of SUL_peak_ (e.g., from baseline to end of therapy in the primary tumor)
SUV_max_(glu)		SUV_max_ corrected for blood glucose level
	tSUV_max_(glu)	SUV_max_(glu) in the primary tumor
SUV_max_(liver)		SUV_max_ corrected for SUV in the liver
	tSUV_max_(liver)	SUV_max_(liver) in the primary tumor
	ΔtSUV_max_(liver)	Change of tSUV_max_(liver) (e.g., from baseline to end of therapy)
	Δtn-meanSUV_max_(liver)	Change of average of SUV_max_(liver)s in primary tumor and regional lymph node metastases (e.g., from baseline to end of therapy)
PET-positive		Presence of PET-vivid lesion
	wbPET-positive	PET-vivid lesions throughout the whole body
	tPET-positive	PET-vivid primary tumor
	nPET-positive	PET-vivid regional lymph node metastases
	mPET-positive	PET-vivid distant metastases
**MTV: Metabolic tumor volume**		Tumor volume defined by FDG–PET; delineation of the tumor volume can be defined with a preset threshold, software based, or it can be determined visually
MTV with fixed threshold		MTV delineated with a fixed threshold
	tMTV2.5	MTV with SUV > 2.5 in the primary tumor
	nMTV2.5	MTV with SUV > 2.5 in regional lymph nodes
	tnMTV2.5	MTV with SUV > 2.5 in the primary tumor and regional lymph nodes
	wbMTV2.5	MTV with SUV > 2.5 throughout the whole body
	ΔtnMTV2.5	Change of tnMTV2.5 (e.g., from baseline to end of therapy)
	tMTV3.0	MTV with SUV > 3.0 in the primary tumor
	wbMTV3.0	MTV with SUV > 3.0 throughout the whole body
	thoracicMTV3.0	MTV with SUV > 3.0 in intrathoracic malignant lesions (lung, pleura, mediastinum)
	ExtrathoracicMTV3.0	Volume with SUV > 3.0 in extrathoracic malignant lesions
	hottest-tumorMTV3.0	MTV with SUV > 3.0 in the hottest tumor throughout the whole body
MTV with relative threshold		MTV delineated with a threshold relative to SUV_max_
	tMTV40	MTV with SUV > 40% of SUV_max_ in the primary tumor
	nMTV40	MTV with SUV > 40% of SUV_max_ in regional lymph node metastases
	mMTV40	MTV with SUV > 40% of SUV_max_ in distant metastases
	wbMTV40	MTV with SUV > 40% of SUV_max_ throughout the whole body
	tMTV42	MTV with SUV > 42% of SUV_max_ in the primary tumor
	tnMTV42	MTV with SUV > 42% of SUV_max_ in the primary tumor and regional lymph node metastases
	wbMTV50	MTV with SUV > 50% of SUV_max_ throughout the whole body
	ΔtnMTV40	Change of MTV with SUV > 40% of SUV_max_ in primary tumor and regional lymph node metastases (e.g., from baseline to end of therapy)
	ΔtnMTV50	Change of MTV with SUV > 50% of SUV_max_ in primary tumor and regional lymph node metastases (e.g., from baseline to end of therapy)
MTV with software-based delineation		MTV delineated by software; studies included all used an isocontouring method with liver as background
	wbMTV_software_	Software-based MTV throughout the whole body
	thoracicMTV_software_	Software-based MTV in all intrathoracic malignant lesions (lung, pleura, mediastinum)
GTV: gross tumor volume		Tumor volume used for radiotherapy planning consisting of regional lymph nodes defined before chemotherapy and tumor volume defined by PET post-chemotherapy
	GTV	
**TLG: Total lesion glycolysis**		Parameter combining FDG uptake and tumor volume; calculated by multiplication of MTV and SUV_mean_ within the MTV
	tTLG2.5	MTV2.5 × SUV_mean_2.5 in primary tumor
	nTLG2.5	MTV2.5 × SUV_mean_2.5 in regional lymph nodes
	tnTLG2.5	MTV2.5 × SUV_mean_2.5 in primary tumor and regional lymph nodes
	wbTLG2.5	MTV2.5 × SUV_mean_2.5 throughout the whole body
	ΔtnTLG2.5	Change of tnTLG2.5 (e.g., from baseline to end of therapy)
	tTLG3.0	TLG3.0 × SUV_mean_3.0 in primary tumor
	wbTLG3.0	TLG3.0 × SUV_mean_3.0 throughout the whole body
	hottest-tumorTLG3.0	TLG3.0 × SUV_mean_3.0 in the hottest tumor throughout the whole body
	tTLG40	MTV40 × SUV_mean_40 in primary tumor
	nTLG40	MTV40 × SUV_mean_40 in regional lymph node metastases
	mTLG40	MTV40 × SUV_mean_40 in distant metastases
	wbTLG40	MTV40 × SUV_mean_40 throughout the whole body
	tTLG42	MTV42 × SUV_mean_42 in primary tumor
	tnTLG42	MTV42 × SUV_mean_42 in primary tumor and regional lymph node metastases
	wbTLG50	MTV50 × SUV_mean_50 throughout the whole body
	wbTLG_software_	MTV_software_ × SUV_mean(software)_ throughout the whole body
	thoracicTLG_software_	MTV_software_ × SUV_mean(software)_ in intrathoracic malignant lesions (lung, pleura, mediastinum)

**Table 2:** Prognostic value of baseline PET parameters.

Study	Patients	Therapy	Endpoints	Univariate Analysis				Multivariate Analysis	
	N (LD/ED)	CCRT/Cht/RT		SUV_max_	Other Uptake Values	MTV	Compound Parameters	PET Parameters	Other Covariates
Özdemir 2020 [25]	153 (153/0)	94/59/0	PFS OS	tSUV_max_: n.s nSUV_max_: n.s.				tSUV_max_: OS nSUV_max_: n.s.	LDH: n.s. Sex: n.s. Albumin: n.s. Cht: regimen: n.s. Treatment response: PFS + OS RT: PFS + OS
	119 (0/119)	0/119/0	PFS OS	tSUV_max_: n.s nSUV_max_: n.s. mSUV_max_: n.s.				tSUV_max_: n.s nSUV_max_: n.s. mSUV_max_: n.s.	LDH: OS Sex: n.s. Albumin: n.s. Cht: regimen: n.s. Treatment response: PFS + OS
Choi 2019 [18]	50 (50/0)	38/11/1	OS	tSUV_max_: OS		tMTV3.0: n.s. wbMTV3.0: OS	tTLG3.0: n.s. wbTLG3.0: OS	tSUV_max_: OS wbMTV3.0: n.s. wbTLG3.0: n.s.	Age n.s. Sex: n.s.
	68 (0/68)	0/65/3	OS	wbSUV_max_: n.s.		hottest-tumorMTV3.0: n.s. wbMTV3.0: OS	hottest-tumorTLG3.0: n.s. wbTLG3.0: OS	wbMTV3.0: OS wbTLG3.0: OS	Age: n.s. LDH: n.s. Sex: n.s.
Kasahara 2019 [19]	98 (40/58)	NA	OS	tSUV_max_: OS LD: tSUV_max_: OS ED: tSUV_max_: n.s.				tSUV_max_: OS LD: tSUV_max_: OS	Stage: OS PS: OS PD-L1: OS
Araz 2019 [26]	38 (15/23)	17/19/0 Sur: 2	OS	wbSUV_max_: n.s	wbSUV_mean(software)_: n.s. wbSUV_peak_: n.s.	wbMTV_software_: OS	wbTLG_software_: n.s.	wbSUV_max_: n.s. wbSUV_mean(software)_: n.s. wbSUV_peak_: n.s. wbMTV_software_: OS wbTLG: n.s.	Age: n.s. LDH: n.s. Sex: n.s.
Chang 2019 [27]	30 (30/0)	30/0/0	PFS OS	tSUV_max_: n.s.	tSUV_max_(glu): PFS + OS	tMTV2.5: OS	tTLG2.5. OS	tSUV_max_(glu): PFS tMTV2.5: OS tTLG: n.s.	None
Fu 2018 [28]	129 (129/0)	129/0/0	PFS OS			wbMTV3.0: PFS + OS		wbMTV3.0: PFS + OS	Age: n.s. Sex: n.s. PS: n.s. Cht regimen: n.s. CTC: PFS + OS
Jin 2018 [16]	46 (46/0)	46/0/0	OS PFS	tSUV_max_: n.s. nSUV_max_: n.s.	tSUV_mean2.5_: n.s. nSUV_mean2.5_: n.s.	tMTV2.5: n.s. nMTV2.5: PFS + OS tnMTV2.5: PFS + OS	tTLG2.5: n.s. nTLG2.5: PFS + OS tnTLG2.5: PFS + OS	nMTV2.5: PFS + OS tnMTV2.5: n.s. nTLG2.5: PFS + OS tnTLG2.5: n.s.	PS: PFS + OS N1 station involvement: n.s. Subcarinal LN metastases: PFS + OS
Kim H 2018 [29]	59 (27/32)	22/37/0	OS PFS	tSUV_max_: n.s.	tSUV_peak_: n.s.	tnMTV2.5: PFS	tnTLG2.5: PFS	tnMTV2.5: n.s. tnTLG2.5: n.s.	Stage: PFS LDH: n.s. RECIST: PFS
Aktan 2017 [20]	46 (46/0)	46/0/0	OS PFS	tSUV_max_: OS nSUV_max_: OS				tSUV_max_: n.s. nSUV_max_: OS	Age: OS
Yilmaz Demirci 2017 [30]	142 (60/82)	38/104/0	OS	tSUV_max_: n.s.				tSUV_max_: n.s.	Stage: n.s. Age: n.s. LDH: OS PS: OS Albumin: OS Calcium: n.s. Thoracic RT: OS PCI: n.s.
Dinc 2016 [31]	90 (33/57)	33/57	OS PFS	tSUV_max_: n.s.				none	Stage: PFS OR: PFS + OS
Kwon 2016 [21]	59 (59/0)	41/14/5 Cht + sur: 4	OS PFS	wbSUV_max_: PFS + OS		wbMTV2.5: PFS + OS	wbTLG2.5: OS + PFS	wbSUV_max_: OS wbMTV2.5: PFS wbTLG2.5: n.s.	Stage: NA ^1^ Age: NA ^1^ LDH: NA ^1^ PS: NA ^1^ ChT (yes vs. no): NA ^1^
Nobashi 2016 [32]	28 (14/14) central SCLC	14/14	OS PFS	tSUV_max_: n.s. wbSUV_max_: n.s.		wbMTV40: PFS + OS	wbTLG40: PFS + OS	tSUV_max_: n.s. wbSUV_max_: n.s. wbMTV40: n.s. wbTLG40: n.s.	Stage: PFS + OS NSE: n.s.
	41 (24/17) peripheral SCLC	13/28	OS PFS	tSUV_max_: n.s. wbSUV_max_: n.s.		wbMTV40: PFS + OS	wbTLG40: PFS + OS	tSUV_max_: n.s. wbSUV_max_: n.s. wbMTV40: PFS + OS wbTLG40: PFS + OS	Stage: OS ^2^ NSE: n.s.
Zer 2016 [33]	55 (24/31)	24/31/0	OS PFS	none ^3^		none ^3^	none ^3^	tSUV_max_: n.s. nSUV_max_: n.s. tMTV42: n.s. tnMTV42: PFS tTLG42: n.s. tnTLG42: OS	Stage: n.s.
Ong 2016 [34]	120 (120/0)	120/0/0	OS DFS LRF DF	tSUV_max_: n.s.	tSUV_mean42_: n.s.	tMTV42: DF	tTLG42: n.s.	tMTV42: n.s.	Stage: DFS + DF Age: DF PS: n.s.
Kim SJ 2015 [15]	82 (31/51) ^4^	31/51	OS PFS	tSUV_max_: n.s. LD: tSUV_max_: n.s. ED: tSUV_max_: n.s.				none	Stage: OS Age: n.s. LDH: OS Sex: n.s. PS: OS
Park 2014 [35]	202 (95/107)	85/117	OS	thoracicSUV_max_: n.s.	thoracicSUV_mean(software)_: n.s.	thoracicMTV_software_: OS LD:thoracic MTV_software_: OS ED: thoracic MTV_software_: n.s.	ThoracicTLG_software_: OS LD: thoracic TLG_software_: OS ED: thoracic TLG_software_: n.s.	thoracicMTV_software_: OS thoracicTLG_software_: OS	Stage: OS Age: OS
Kim MH 2014 [14]	114 (26/88) ^4^	CCRT or Cht: 114	OS PFS	tSUV_max_: n.s.	Wb-meanSUV_max_: n.s.		wb-sumSUV_max_: OS + PFS LD: wb-sumSUV_max_: PFS ED: wb-sumSUV_max_: OS + PFS	wb-sumSUV_max_: PFS + OS	Stage: n.s. Age: OS LDH: n.s. Sex: PFS Cht (no. of cycles): PFS + OS OR: PFS + OS NSE: n.s. CYFRA21-1: n.s.
Lee J 2014 [36]	41 (41/0)	41/0/0	OS PFS		tSUV_max_(liver): OS			tSUV_max_(liver): OS	LDH: PFS + OS Sex: OS OR: OS
Go 2014 [37]	145 (61/84)	44/101	OS PFS	wbSUV_max_: n.s.	Wb-meanSUV_max_: n.s.		wb-sumSUV_max_^5^: PFS + OS	wb-sumSUV_max_^5^: PFS + OS	Stage: PFS Sex: PFS OR: PFS No. of lesions: PFS
Inal 2013 [38]	54 (24/30)	24/30	OS	tSUV_max_: n.s.				none	Stage: OS PS: OS DM: n.s.
Gomez 2014 [17]	50 (50/0)	50/0/0	OS	tSUV_max_: n.s. nSUV_max_: n.s.	tn-meanSUV_max_: n.s.				
Oh 2013 [13]	91 (0/91) ^6^	26/65	OS PFS	wbSUV_max_: n.s. thoracicSUV_max_: n.s. extrathoracicSUV_max_: n.s.		wbMTV3.0: OS + PFS thoracicMTV3.0: n.s. extrathoracicMTV3.0: PFS + OS		wbMTV3.0: n.s. extrathoracic MTV3.0: PFS	Age: n.s. PS: OS Cht (no. of cycles): PFS + OS RT: n.s. PCI: n.s. Bone mets: n.s. Liver mets: n.s. No. of extrathoracic foci: OS
Jhun 2013 [39]	246 (NA) ^7^	NA ^7^	OS	tSUV_max_: n.s.				none	Stage: OS Age: OS LDH: OS PS: OS Albumin: n.s.
Oh 2012 [12]	106 (45/61) ^6^	45/61/0	PFS OS	wbSUV_max_: n.s.		wbMTV3.0: PFS + OS LD: wbMTV3.0: PFS + OS ED: wbMTV3.0: PFS + OS		wbSUV_max_: n.s. wbMTV3.0: PFS + OS	Stage: OS + PFS LDH: n.s. PS: n.s. Cht (no. of lines): n.s.
Van der Leest 2012 [22]	75 (35/40)	26/28/0 sur: 4 None: 13 NA: 4	OS PFS	tSUV_max_: n.s. LD: tSUV_max_: n.s. ED: tSUV_max_: OS + PFS					
Zhu 2011 [23]	98 (41/57)	57/41	OS PFS	tSUV_max_: PFS + OS	wb-meanSUV_mean2.5_: PFS + OS	wbMTV2.5: PFS + OS LD: wbMTV2.5: PFS + OS ED: wbMTV2.5: PFS + OS	wbTLG2.5: PFS +OS LD: wbTLG2.5: PFS +OS ED: wbTLG2.5: PFS +OS	tSUV_max_: n.s. wb-meanSUV_mean2.5_: n.s. wbMTV2.5: PFS + OS wbTLG2.5: PFS + OS	Stage: OS + PFS LDH: OS + PFS
Lee YJ 2009 [40]	76 (41/35)	41/35	OS PFS	tSUV_max_: NA ^3^ wbSUV_max_: NA ^3^	wb-meanSUV_max_^8^: OS + PFS			wb-meanSUV_max_^8^: PFS + OS tSUV_max_: n.s. ^9^ wbSUV_max_: n.s. ^9^	Stage: OS + PFS LDH: PFS PS: OS
Chong 2007 [24]	15 (9/6)	NA	OS	wbSUV_max_: OS ^10^					
Pandit 2003 [41]	8 (4/4)	NA	OS	wbSUV_max_: n.s.	PET-positive: n.s.				

^1^ Kwon et al. did not provide results from multivariate analysis of non-PET-parameters; ^2^ stage was independently prognostic in multivariate analysis including SUV_max_, not when including MTV or TLG; ^3^ results from multivariate analysis available only; ^4^ overlapping cohorts of Kim SJ and Kim MH; ^5^ Sum of SUV_max_ in 1-5 lesions identified by RECIST; ^6^ overlapping cohorts in the two studies by Oh; ^7^ data only available for a larger cohort of 320 patients. Mixed stage and mixed treatments. ^8^ mean of SUV_max_ in all lesions, however one lesion per organ only; ^9^ The model for multivariate analysis of tSUV_max_ and wbSUV_max_ was not described, neither was results from other included covariates; ^10^ Raw data available, prognostic value was calculated with cut-off suggested by authors; N: number; LD: limited disease; ED: extensive disease; CCRT: concomitant chemo-radiotherapy; Cht: chemotherapy; RT: radiotherapy; SUV: standardized uptake value; MTV: metabolic uptake value; PFS: progression free survival; OS: overall survival; t: (prefix) within primary tumor; n.s.: non-significant; n: (prefix) within n-sites; LDH: blood-lactate dehydrogenase; m: (prefix) within m-sites; wb: (prefix) wholebody; TLG: total lesion glycolysis; NA: not available; PS: performance status (WHO or Karnofsky’s); PD-L1: programmed death ligand-1; sur: surgery; SUV_max_(glu): SUV_max_ corrected for blood glucose level; CTC: circulating tumor cells; tn: (prefix) within primary tumor and n-sites; LN: lymph nodes; RECIST: response evaluation criteria in solid tumors; PCI: prophylactic cranial irradiation; OR: objective response; NSE: Neuron-specific enolase; DFS: disease free survival; LRF: loco-regional failure; DF: distant failure; SUV_max_(liver): SUV_max_ corrected for SUV_max_ in the liver; DM: diabetes mellitus; mets: metastases; no: number.

**Table 3:** Prognostic value of post-treatment PET parameters.

Study	Patients				Univariate Analysis			Multivariate Analysis	
	N (LD/ED)	Therapy CCRT/Cht/RT	Timing of PET (Interval from End of Treatment)	Endpoints	SUV_max_	Other Uptake Values	MTV and TLG	PET Parameters	Other Covariates
Quartuccio 2019 [42]	164 (NA/NA)	62/89/13	<3 months	PFS OS	tSUV_max_: n.s. nSUV_max_: n.s. mSUV_max_: n.s.	tSUV_mean40_: n.s. nSUV_mean40_: n.s. mSUV_mean40_: n.s. tPET-positive: n.s. nPET-positive: n.s. mPET-positive: PFS + OS	tMTV40: n.s. nMTV40: n.s. mMTV40: n.s. tTLG40: n.s. nTLG40: n.s. mTLG40: n.s.	NA	NA
Kim H 2018 [29]	59 (27/32)	22/37/0	0.5–2.7 months	OS PFS	tSUV_max_: OS + PFS	tSUV_peak_: OS + PFS	tnMTV2.5: PFS + OS tnTLG2.5: OS + PFS	tSUV_peak_: n.s. tnMTV2.5: PFS	Stage: PFS LDH: n.s. RECIST: PFS
Lee J 2014 [36]	41 (41/0)	41/0/0	3 weeks	OS PFS		tSUV_max_(liver) ^1^: n.s.		none	Sex: OS LDH: PFS + OS OR: OS
Ziai 2013 [43]	29 (13/16)	21/8/0	4.3–7.5 months (from baseline PET)	PFS OS	^2^ SUV_max_: PFS + OS	Wb-sumSUL_peak_^3^: PFS + OS wbPET-positive ^4^: PFS + OS		^2^ SUV_max_: n.s. Sum-wbSUL_peak_^3^: OS wbPET-positive ^4^: PFS + OS	Presence of mets: n.s.
Onitilo 2008 [44]	22 (22/0)	17/5/0	<4 months	PFS OS		wbPET-positive (<2.5 and visually corrected): PFS		NA	NA
Blum 2004 [45]	25 (NA/NA)	NA	NA ^5^	TTP		wbPET-positive: longer median TTP (no statistical analysis)		NA	NA
Pandit 2003 [41]	38 (24/13) NA:1	23/14/1	4 days–48 months (54 PETs included)	OS	wbSUV_max_: OS	wbSUV_mean_^6^: n.s. wbPET-positive: OS		NA	NA

^1^ SUV_max_ corrected for SUV_max_ in the liver; ^2^ anatomical limitation not specified; ^3^ sum of SUL_peak_ in 1–5 lesions; ^4^ defined as visible uptake vs. no visible uptake (CMR vs. non-CMR); ^5^ detection of residual disease after therapy or suspected recurrence. ^6^ delineation method for the ROI/MTV not specified; TTP: time to progression.

**Table 4:** Prognostic value of PET parameter change, early and final response evaluation. All PET parameters were compared with the baseline PET parameter.

Study	Patients				Univariate Analysis		Multivariate Analysis	
	N (LD/ED)	Therapy: CCRT/Cht	Timing of Response Evaluation	Endpoints	ΔSUV	ΔMTV and ΔTLG	PET Parameters	Other Covariates
Kim H 2018 [29]	59 (27/32)	22/37	Final response: 0.5–2.7 months after therapy	OS PFS	ΔtSUV_max_: OS + PFS ΔtSUV_peak_: OS + PFS	ΔtnMTV2.5: PFS ΔtnTLG2.5: n.s.	ΔtSUV_peak_: OS	Stage: PFS LDH: n.s. RECIST: PFS
Lee J 2014 [36]	41 (41/0)	41/0	Final response: 3 weeks after end of CCRT	OS PFS	ΔtSUV_max_(liver) ^1^: n.s Δtn-meanSUV_max_(liver) ^1^: OS + PFS		ΔtSUV_max_(liver) ^1^: n.s Δtn-meanSUV_max_(liver) ^1^: PFS ^2^	Sex: OS LDH: PFS + OS OR: OS
Ziai 2013 [43]	29 (13/16)	21/8	Final response: 4.3–7.5 month from baseline-PET	PFS OS	ΔtSUL_peak_^3^: PFS		None	Presence of mets: PFS
V Loon 2011 [46]	15 (15/0)	15/0	Early response: after 1 cycle Cht	OS		ΔtnMTV40: OS ΔtnMTV50: OS	NA	NA

^1^ SUV_max_ corrected for SUV_max_ in the liver; ^2^ larger reduction associated with lower HR (i.e., longer PFS); ^3^ response by PERCIST categorized in CMR (visual disappearance of all metabolically active tumor) vs. PMR + SMD (<30% increase of SUL_peak_ or reduction of SUL_peak_) vs. PMD (>30% increase in SUL_peak_). The study obtained identical results from response by the European Organization for Research and Treatment of Cancer (EORTC) criteria. Δ: delta: the change of a parameter from baseline; SUL: SUV corrected for lean body mass; PERCIST: PET response criteria in solid tumor; CMR: complete metabolic response; PMR: partial metabolic response; SMD: stable metabolic disease; PMD: progressive metabolic disease; NA: not available.

**Table 5:** Prognostic value of PET parameters in studies with PET at mixed treatment phases.

Study	Patients				Univariate Analysis			Multivariate Analysis	
	N (LD/ED)	Therapy CCRT/Cht	Timing of PET	Endpoints	SUV	MTV	TLG	PET Parameters	Other Covariates
Mirili 2019 [47]	54 (16/36)	19/26 No therapy: 9	Baseline or after therapy (not further specified)	OS PFS	tSUV_max:_ OS tSUV_mean40_: n.s.	tMTV40: PFS + OS wbMTV40: PFS + OS	tTLG40 n.s. wbTLG40: PFS + OS	wbTLG40: n.s.	Age: OS Stage: OS Sex: n.s. NLR: OS
Reymen 2013 [48]	119 (119/0)	119/0	Baseline/during therapy ^1^	OS		GTV: OS		GTV: OS	PS: OS Stage: n.s. Age: n.s. Sex: n.s. LDH: n.s. N-status: n.s. SER: n.s.
Arslan 2011 [49]	25 (10/15)	NA	Baseline (12) or restaging/response evaluation (13)	OS	wbSUV_max_: n.s. wbSUV_mean2.5:_ n.s.	wbMTV2.5: n.s. wbMTV50: n.s.	wbTLG2.5: n.s. wbTLG50:OS	wbSUV_max_: n.s. wbSUV_mean2.5:_ n.s. wbMTV2.5: n.s. wbMTV50: n.s. wbTLG2.5: n.s. wbTLG50: OS	Baseline vs. restaging: n.s.

^1^ Post-chemotherapy tumor volume and pre-chemotherapy nodal volume. NLR: neutrophil/lymphocyte ratio; GTV: gross tumor volume consisting of post-chemotherapy tumor volume and pre-chemotherapy nodal volume; SER: time from start of any therapy to end of radiotherapy.

## Supplementary Materials

The following are available online at https://www.mdpi.com/2075-4418/11/2/174/s1: Supplementary File S1: Table S1: Studies included in meta-analysis. Definitions of PET parameters and cutoff. Supplementary File S2: Techniques used in meta-analysis when estimate and standard error (SE) were not directly available.
