# Supplementary material for: Prognostic Value of 18F–FDG–PET Parameters in Patients with Small Cell Lung Cancer: A Meta-Analysis and Review of Current Literature
Source: Diagnostics (Basel). 2021 Jan 26;11(2):174. doi: 10.3390/diagnostics11020174 (PMC7912276; doi:10.3390/diagnostics11020174)
Supplement: Supplementary file 1 [file diagnostics-11-00174-s001.zip › diagnostics-1045395-supplementary/diagnostics-1045395-File SI+File S2/diagnostics-1045395-File S1.pdf]

Table S1: Studies included in meta-analysis. Definition of PET-parameters and cutoff.

Study	Patients		SUV_{max}				MTV			
	N (LD/ED)	Cutoff type	definition	Cutoff value	Endpoints		definition	Cutoff value	Endpoints	
					OS	PFS			OS	PFS
Choi (1)	118 (50/68)	Continuous or optimal	LD: tSUVmax ED: wbSUVmax		+		wbMTV3.0[1]		+	
Kasahara (2)	98 (40/58)	Optimal	$tSUV_{max}$	5.1	+					
Araz (3)	38 (15/23)	Median					$wbMTV_{software}$	147	+	
Chang (4)	30 (30/0)	75th percentile					tMTV2.5	166.6	+	
Fu (5)	129 (129/0)	Median					wbMTV3.0	266.5	+	+
Jin (6)	46 (46/0)	Optimal	$tSUV_{max}$	16	+	+	tMTV2.5	68	+	+
Aktan (7)	46 (46/0)	Optimal	$tSUV_{max}$	9.3	+	+				
Dinc (8)	90 (33/57)	Optimal	$tSUV_{max}$	10		+				
Yilmas (9)	142 (60/82)	Continuous	$tSUV_{max}$		+					
Kwon (10)	59 (59/0)	Continuous	$wbSUV_{max}$		+	+	wbMTV2.5		+	+
Nobashi1 (11)	28 (14/14)	Median	$tSUV_{max}$[2]	10.6	+	+	wbMTV40	43.9	+	+
Nobashi2 (11)	41 (24/17)	Median	$tSUV_{max}$[2]	9.3	+	+	wbMTV40	38.3	+	+
Kim SJ (12)	82 (31/51)	Median	$tSUV_{max}$	8.2	+	+				
Ong (13)	120 (120/0)	Median	$tSUV_{max}$	12.15	+	+	tMTV42	21.45	+	+
Park (14)	202 (95/107)	Optimal					$thoracicMTV_{software}$	100	+	
Go (15)	145 (61/84)	Median	$wbSUV_{max}$	8.29	+					
Gomez (16)	50 (50/0)	Median	$tSUV_{max}$	12.7	+					
Oh 2012 (17)	106 (45/61)	Median	$wbSUV_{max}$	10.4	+	+	wbMTV3.0	127	+	+
Zhu (18)	98 (41/57)	Optimal	$tSUV_{max}$	7.8	+	+	wbMTV2.5	64.4	+	+
Chong (19)	15 (9/6)	Optimal	$wbSUV_{max}$	13.7	+					

[1]Results from tMTV3.0 and wbMTV3.0 were available from the LD-cohort. wbMTV3.0 was included in the meta-analysis, as wbMTV was more frequently presented in the remaining studies, in order to reduce interstudy heterogeneity.

[2]Results from $tSUV_{max}$ and $wbSUV_{max}$ were available. $tSUV_{max}$ was included in the meta-analysis, as tSUVmax was more frequently used in the remaing studies, in order to reduce interstudy heterogeneity.
