# Supplementary material for: Prognostic Value of 18F–FDG–PET Parameters in Patients with Small Cell Lung Cancer: A Meta-Analysis and Review of Current Literature
Source: Diagnostics (Basel). 2021 Jan 26;11(2):174. doi: 10.3390/diagnostics11020174 (PMC7912276; doi:10.3390/diagnostics11020174)
Supplement: Supplementary file 1 [file diagnostics-11-00174-s001.zip › diagnostics-1045395-supplementary/diagnostics-1045395-File SI+File S2/diagnostics-1045395-File S2.pdf]

Techniques used in meta-analysis when estimate and SE were not directly available

Reconstructing survival data

In a number of studies (MTVOS: Araz et al. [1] and Chang et al. [2]; SUVOS and SUVPFS: Aktan et al. [3]) and Kwon et al. [4], the published Kaplan-Meier curves allowed reconstruction of the full set of survival data (i.e., both times of events, OS or PFS, and times of censoring). Thereby, data could be re-analyzed to obtain estimates of hazard ratio and its standard error.

Reconstructing survival curves

In a number of studies (MTVOS: Choi et al. (SCLC-ED) [5]; SUVOS: Go et al. [6] and Choi et al. (SCLC-LD) [5]; SUVPFS: Dinc et al. [7]), Kaplan-Meier curves were published that allowed reconstruction of the survival *distribution*, albeit not the complete set of observed times of events and censorings. Thereby, it was possible to simulate observations from the relevant distributions and estimate the hazard ratio. Furthermore, from the p-values for analyses where MTV or SUV were used as a quantitative covariate the standard error, SE, was estimated from the equation

$$p = P\left(\chi_1^2 > (\frac{\log(HR)}{SE})^2\right).$$

Reconstructing individual values of SUV and MTV

In the paper by Kwon et al. [4], a scatterplot of MTV versus SUV was presented from which the individual values, say x, could be read. The hazard ratio from a comparison of survival curves for patients with values of x (=MTV or SUV) below or above the median, M was then obtained as

$$HR = \frac{1}{3}\sum_t \frac{\log(S_+(t))}{\log(S_-(t))},$$

where t was chosen to get a baseline survival function S0(t) of 0.3, 0.5, or 0.7. Here

$$S_+(t) = \frac{1}{n/2}\sum_{i:x_i \geq M} S_0(t)^{\exp(\widehat{\beta}x_i)}, \quad S_-(t) = \frac{1}{n/2}\sum_{i:x_i < M} S_0(t)^{\exp(\widehat{\beta}x_i)}$$

and exp(β) is the hazard ratio when using x as quantitative covariate (given in the paper). Finally, the standard error of the HR was estimated using again the first equation for p. Robustness to the choices of t was investigated.

Reconstructing hazard ratios for high vs. low values from analyses with quantitative covariates

In a number of studies (MTVOS: Choi et al. (SCLC-LD) [5]; SUVOS: Yilmaz Demirci et al. [8], Choi et al. (SCLC-ED) [5]), only the hazard ratio and SE when using the covariate x(=MTV or SUV) were given, together with the sample size, n, and median, M and range, say x(1) to x(n) for x. Since the distributions of MTV and SUV tended to be right-skewed, they distribution of y=log(x) was approximated by a normal distribution with mean log(M) and some SD. For a sample of size n from a normal distribution with mean μ and SD σ, the expected value of the r'th smallest observation can be approximated by

$$E(y_{(r)}) = \mu + \Phi^{-1}\left(\frac{r - \frac{\pi}{8}}{n + 1 - \frac{2\pi}{8}}\sigma\right)$$

Using Blom G: 'Statistical estimates and transformed beta-variables' [9] for the minimum and maximum values (r=1 or r=n), the standard deviation, σ, can be estimated. Knowing the standard deviation, the expected value of y given above μ is

$$E(y \mid y > \mu) = \mu + \sigma\sqrt{\frac{2}{\pi}},$$

and, similarly the expected value given below the mean is the same formula with a '-' instead of a '+'. The difference between the expected values of x given above or below the median can, thus, be approximated by

$$D = M\exp(\sigma\sqrt{2/\pi}) - M/\exp(\sigma\sqrt{2/\pi})$$

and the log(HR) for comparing patients with values above and below the median is finally approximated by

Dβ with a standard error of D·SE, where exp(β) is the hazard ratio when using x as a quantitative covariate and SE its standard error (possibly calculated from a 95% confidence interval for exp(β)).

1. Araz M, Soydal C, Ozkan E, Sen E, Nak D, Kucuk ON et al. Prognostic value of metabolic parameters on baseline 18F-FDG PET/CT in small cell lung cancer. Q J Nucl Med Mol Imaging. 2019. doi:10.23736/S1824-4785.19.03169-8.

2. Chang H, Lee SJ, Lim J, Lee JS, Kim YJ, Lee WW. Prognostic significance of metabolic parameters measured by (18)F-FDG PET/CT in limited-stage small-cell lung carcinoma. J Cancer Res Clin Oncol. 2019. doi:10.1007/s00432-019-02848-9.
3. Aktan M, Koc M, Kanyilmaz G, Yavuz BB. Prognostic value of pre-treatment (18)F-FDG-PET uptake in small-cell lung cancer. Ann Nucl Med. 2017;31(6):462-8. doi:10.1007/s12149-017-1178-z.
4. Kwon SH, Hyun SH, Yoon JK, An YS, Oh YT, Choi JH et al. The Highest Metabolic Activity on FDG PET Is Associated With Overall Survival in Limited-Stage Small-Cell Lung Cancer. Medicine. 2016;95(5):e2772. doi:10.1097/MD.0000000000002772.
5. Choi EK, Park M, Im JJ, Chung YA, Oh JK. Prognostic value of (18)F-FDG PET/CT metabolic parameters in small cell lung cancer. J Int Med Res. 2019:300060519892419. doi:10.1177/0300060519892419.
6. Go SI, Song HN, Kang JH, Kang MH, Kim MJ, Jung J et al. The clinical impact of the sum of the maximum standardized uptake value on the pretreatment with F-FDG-PET/CT in small-cell lung cancer. Oncology. 2014;86(1):1-9. doi:10.1159/000357136.
7. Dinc NS, Aydin K, Odabas H, Ercelep O, Tufan G, Seker M et al. Pretreatment PET/CT Standardized Uptake Values Play a Role in Predicting Response to Treatment and Survival in Patients with Small Cell Lung Cancer. Oncol Res Treat. 2016;39(3):130-4. doi:10.1159/000444272.
8. Yilmaz Demirci N, Yilmaz U, Biner Uslu I, Dikmen A, Yilmaz A, Erdogan Y. Prognostic significance of standardised uptake value (SUVmax) measured on 18F-fluorodeoxyglucose positron emission tomography/computed tomography in patients with small cell lung cancer. European journal of cancer care. 2017;26(5). doi:10.1111/ecc.12485.
9. Blom G. Statistical estimates and transformed beta-variables. Wiley, New York, USA. 1954.
